# Microglia Morphological Categorization in a Rat Model of Neuroinflammation by Hierarchical Cluster and Principal Components Analysis

**DOI:** 10.3389/fncel.2017.00235

**Published:** 2017-08-08

**Authors:** María del Mar Fernández-Arjona, Jesús M. Grondona, Pablo Granados-Durán, Pedro Fernández-Llebrez, María D. López-Ávalos

**Affiliations:** Departamento de Biología Celular, Facultad de Ciencias, Genética y Fisiología, Instituto de Investigación Biomédica de Málaga (IBIMA), Universidad de Málaga Málaga, Spain

**Keywords:** fractal, microglia, morphofunctional, morphometric, neuraminidase

## Abstract

It is known that microglia morphology and function are closely related, but only few studies have objectively described different morphological subtypes. To address this issue, morphological parameters of microglial cells were analyzed in a rat model of aseptic neuroinflammation. After the injection of a single dose of the enzyme neuraminidase (NA) within the lateral ventricle (LV) an acute inflammatory process occurs. Sections from NA-injected animals and sham controls were immunolabeled with the microglial marker IBA1, which highlights ramifications and features of the cell shape. Using images obtained by section scanning, individual microglial cells were sampled from various regions (septofimbrial nucleus, hippocampus and hypothalamus) at different times post-injection (2, 4 and 12 h). Each cell yielded a set of 15 morphological parameters by means of image analysis software. Five initial parameters (including fractal measures) were statistically different in cells from NA-injected rats (most of them IL-1β positive, i.e., M1-state) compared to those from control animals (none of them IL-1β positive, i.e., surveillant state). However, additional multimodal parameters were revealed more suitable for hierarchical cluster analysis (HCA). This method pointed out the classification of microglia population in four clusters. Furthermore, a linear discriminant analysis (LDA) suggested three specific parameters to objectively classify any microglia by a decision tree. In addition, a principal components analysis (PCA) revealed two extra valuable variables that allowed to further classifying microglia in a total of eight sub-clusters or types. The spatio-temporal distribution of these different morphotypes in our rat inflammation model allowed to relate specific morphotypes with microglial activation status and brain location. An objective method for microglia classification based on morphological parameters is proposed.

**Main points**

- Microglia undergo a quantifiable morphological change upon neuraminidase induced inflammation.
- Hierarchical cluster and principal components analysis allow morphological classification of microglia.
- Brain location of microglia is a relevant factor.

## Introduction

Microglial cells are resident macrophages of myeloid origin in the central nervous system, which were introduced by Pío del Río-Hortega (Río-Hortega, 1919a,b,c,d; Sierra et al., 2016). He described the distribution and morphological phenotype of microglia and also recognized that ramified cells transformed into rod or amoeboid microglia in different situations of brain disease or pathology. In the normal brain they have been considered as “resting microglia”, but recent findings point that microglia are the most sensitive sensors in the brain, constantly scanning the parenchyma environment as surveillant cells (Nimmerjahn et al., 2005). Upon the detection of any brain lesion or dysfunction, microglial cells acquire an “activated” state which displays inflammatory and phagocytic features. Thus, they are the first line of defense within the brain, and are considered as the major orchestrators of the brain inflammatory response (Nimmerjahn et al., 2005; Kettenmann et al., 2011, 2013; Gomez-Nicola and Perry, 2015).

The morphology of microglia is one of its more outstanding characteristics. Although these cells are apparently evenly distributed in the nervous parenchyma, more detailed observations revealed that neither the morphology nor the distribution are equal in all brain locations, providing evidence that microglial cells are sensitive to the surrounding microenvironment. According to their shape, microglial cells have been categorized into three broadly distinct subtypes: compact, longitudinally branched and radially branched (Lawson et al., 1990). These morphologies are closely related to their functional state (Davis et al., 1994). Under basal healthy conditions, a resting phenotype characterized by a ramified morphology predominates. However these microglia are not actually resting but continuously scanning their environment (Nimmerjahn et al., 2005; Olah et al., 2011), pruning synapses and regulating neuronal activity, providing a “fine-tuning” of neural circuits (Paolicelli et al., 2011; Schafer et al., 2012; Miyamoto et al., 2013) and neurotransmitter signaling/synaptic transmission (Li et al., 2012; Béchade et al., 2013; Domercq et al., 2013).

In situations of neuroinflammation or after injury, a stepwise de-ramification of microglia has been repeatedly observed. Thus, ramified microglia can transform into an “activated state”, characterized by swollen ramified cells with a larger cell body and shorter, thick processes, or alternatively microglia can adopt a “reactive state”, typically small, spherical cells, but can also exhibit rod-shape or amoeboid-like morphologies (Davis et al., 1994). A further state can be a phagocytic one, presenting a “reactive phenotype” with processes containing pyknotic fragments. This state is observed in pathological situations (Streit et al., 1999), but also in physiological conditions during brain development or in neurogenic niches (Sierra et al., 2013). Some microglial morphologies are associated to a motility stage, where cells present dynamic processes that exhibit cycles of extension and retraction, or even a locomotory stage, where microglia actually move to another location within the tissue (Stence et al., 2001; Petersen and Dailey, 2004).

At the same time, increasing evidence suggests different microglia phenotypes from the inflammatory point of view. Upon infection or injury, microglial cells are polarized to the pro-inflammatory phenotype (M1) producing cytokines such as TNF-α and IL-1β. This state is associated with damages induced by inflammation. Also, an alternative anti-inflammatory phenotype (M2) may arise, in which microglial cells express IL-10 and TGF-β, and are prone to remove cellular debris and promote tissue repair (Olah et al., 2011; Walker et al., 2014; Orihuela et al., 2016).

With the aim of better understanding the mechanisms related to the inflammatory role of microglia, several studies have tried to associate the morphological changes described above with their physiological or pathological role in the brain. Different morphological parameters of microglia have been quantified in physiological conditions in healthy brains, both in rats and humans (Kongsui et al., 2014; Torres-Platas et al., 2014). Besides, the shape of microglial cells has been analyzed in pathological situations such as cranial injuries (Soltys et al., 2001; Zanier et al., 2015), under conditions of physiological (salt load) or psychological stress (Ayoub and Salm, 2003; Hinwood et al., 2013) and in mouse models of Alzheimer’s disease (Baron et al., 2014). A particular and enriching kind of morphological analysis is that performed on cultured microglia (Glenn et al., 1992; Bernhardi and Nicholls, 1999; Amadio et al., 2013; McWhorter et al., 2013). Moreover, plasticity and mobility of microglia have been explored (Madore et al., 2013; Hefendehl et al., 2014; Eyo et al., 2016; Kapoor et al., 2016).

Some studies went further and tried to establish different types of microglial cells based on the quantification of morphological parameters. Some statistical procedures, such as PCA, can detect small cell changes (Soltys et al., 2005) or, in the case of hierarchical cluster analysis (HCA), may allow to put forward a new microglia classification. In this way, new microglia categorizations have been proposed in different experimental paradigms, such as visual learning in monkeys (Santos-Filho et al., 2014), hypoglossal axotomy (Yamada and Jinno, 2013), experimental infections (de Sousa et al., 2015; Diniz et al., 2016), and a mouse model of amyotrophic lateral sclerosis (Ohgomori et al., 2016). These works objectively proposed up to four microglial cells types based on morphological characteristics. These morphotypes has been associated to a physiological/pathological spatiotemporal condition; problems related to an arbitrary or subjective classification are therefore avoided.

The aim of the present study was to establish an unbiased classification of the microglial cells present in an acute neuroinflammatory process, specifically that triggered by a single intracerebroventricular injection of the enzyme neuraminidase (NA; Grondona et al., 1996; Granados-Durán et al., 2015). NA is an enzyme found in the surface of certain bacteria and virus, which cleaves glycosidic linkages of sialic acid. The administration of NA into the lateral ventricle (LV) provokes an aseptic neuroinflammation process, which subsides about 2 weeks later. NA is distributed by the CSF flow, so inflammation first affects choroid plexus and brain regions nearby the ventricles such as the septofimbrial nucleus, and later reaches the hypothalamus and other more distant areas such as the hippocampus. Microglial cells present in these regions are susceptible of activation, by a so far unknown mechanism. In the current study, first we searched for significant morphological differences between microglial cells present in different brain areas, or at different times after NA/saline injection. Next, a HCA was performed to come up with a possible number of clusters. A linear discriminant analysis (LDA) allowed determining which morphological parameters were more relevant and appropriate to classify microglia in the different clusters, in particular under acute inflammation. With the purpose of unravelling new microglia subtypes, a further PCA was accomplished. As a result, a logical decision tree was proposed, which allowed the allocation of microglial cells in our model to a particular morphotype according to their parameter values.

## Materials and Methods

### Animals

Male Wistar rats (350 g) were provided by Charles River Laboratories (Barcelona, Spain). An acute neuroinflammatory process was generated in these animals by a single injection of the enzyme NA within the right LV of the rat brain (Grondona et al., 1996; Granados-Durán et al., 2015). Sham rats were injected with 0.9% sterile saline. Rats were sacrificed at the following times after injection: 2, 4 and 12 h. The number of replicas was *n* = 5 in the case of the experimental groups, and *n* = 3 for sham groups, per each experimental time.

Animals were maintained on a 12 h light/dark cycle, at 23°C and 60% humidity, with food and water available *ad libitum*. Animal care was performed according to guidelines established by Spanish legislation (RD 53/2013) and the European Union regulation (2010/63/EU). All procedures performed were approved by the ethics committee of Universidad de Málaga (Comité Ético de Experimentación de la Universidad de Málaga; reference 2012-0013). All efforts were made to minimize the number of animals used and their suffering.

### Intracerebroventricular Injection

Animals were anesthetized with ketamine/xylazine solution (80 and 12 mg/kg, respectively; Sigma-Aldrich) and positioned in a stereotaxic frame. A scalp incision along the sagittal midline was performed to access the skull and the bone was perforated with a drill in the following coordinates: 0.5 mm posterior and 1.4 mm lateral from Bregma (Paxinos and Watson, 2007). NA from *Clostridium perfringens* (Roche Diagnostics, Basel, Switzerland, ref. 11 585 886 001) dissolved in 0.9% sterile saline was administered by a single injection 3.5 mm below the dura mater into the right lateral cerebral ventricle. With the aid of a pump, 500 mU (in 20 μL) of NA were perfused for 10 min with a rate of 2 μL/min.

### Brain Tissue Preparation and Immunohistochemistry

Prior to sacrifice, the animals were anesthetized again and systemically perfused with 0.9% saline, followed by 4% parafolmaldehyde. Brains were removed and post-fixed overnight in the same fixative solution. They were later sectioned with a vibratome (40 μm thickness) in the coronal plane, and the sections stored in 0.1 M phosphate buffered saline (PBS) with 0.02% azide. Three brain sections per animal, including the lateral venticles, the third and the fourth ventricles respectively (approximate distance from Bregma −0.80 mm, −3.30 mm and −11.50 mm), were selected for immunohistochemistry. Free floating sections were first treated to inhibit/quench endogenous peroxidase with 10% methanol and 3% hydrogen peroxide in PBS during 45 min. After washings with PBS, nonspecific binding sites were saturated with PBT solution (0.3% bovine serum albumin, 0.3% Triton X-100 in PBS pH 7.3). The primary antibodies used were rabbit polyclonal anti-IBA-1 (1:1000; Wako) to label macrophages/microglial cells, and goat polyclonal anti-IL-1β (1:500; R&D Systems) to target M1 activated microglia/macrophages. Primary antibodies were incubated overnight at 4°C. The following morning the sections were washed and incubated with biotinylated secondary antibody (goat anti-rabbit 1:500 from Pierce, or horse anti-goat 1:1000 from Vector) at room temperature for 1.5 h. The avidin-biotin-complex amplification system (ABC; 1:250; Thermo Fisher Scientific) was later employed (room temperature, 45 min) to detect the secondary biotinylated antibodies. The peroxidase activity was revealed with 0.05% diaminobenzidine and 0.03% hydrogen peroxide in PBS for 10 min. After thorough washes, the sections were then mounted onto gelatin-coated slides, air dried, dehydrated in graded ethanol, cleared in xylene, and coverslipped with Kukitt mounting medium.

Colocalization of IBA-1 and IL-1β label was performed by double immunofluorescence using the same primary antibodies, which were incubated simultaneously. In this case the secondary antibodies were goat anti-rabbit Alexa 488 (1:1000; Molecular Probes) and donkey anti-goat Alexa 594 (1:1000; Invitrogen). Samples were mounted onto gelatin-coated slides, coverslipped with the anti-fading agent Mowiol 4–88 (Calbiochem/EMD Chemicals) and stored at 4°C. Negative controls of the immunohistochemistry consisted in omitting the primary antibodies.

### Image Acquisition

Image acquisition was carried out with the aim of morphometric analysis of microglial cells. For this purpose digital color images of tissue sections DAB-stained with IBA-1 antibody were obtained using an Olympus VS120 microscope. The UPLSAPO 60×O oil immersion objective was used to capture high resolution images (pixel size = 115 nm^2^) of the selected areas. A multi-plane virtual-Z mode allowed to capture 20 images (1 μm thick) in 20 μm depth of the tissue section, which were later combined to obtain a single high quality image including detailed magnification of ramified processes of the cells. The three areas studied were scanned in two sections per animal. Each acquired image was a TIFF file of 96 ppi, and contained at least 30 cells. These images were cropped to delimit single cells as is later described in *Image processing*.

Moreover, with the aim of describing the progression of the acute inflammation, a second set of images was acquired from tissue sections DAB-stained with anti-IL-1β. The three areas selected were scanned to establish the spatio-temporal spreading of the neuroinflammation, using in this case the UPLSAPO 40× 2 objective.

For immunofluorescence colocalization of IBA-1 and IL-1β, images were acquired using the inverted microscope LEICA SP5 II equipped with a confocal scan unit. Images were captured with 63× objective using Ar (488 nm) and He-Ne (594 nm) lasers. Three-dimensional projections were obtained by means of the *z*-stack mode.

### Image Processing

**Figure 1:**
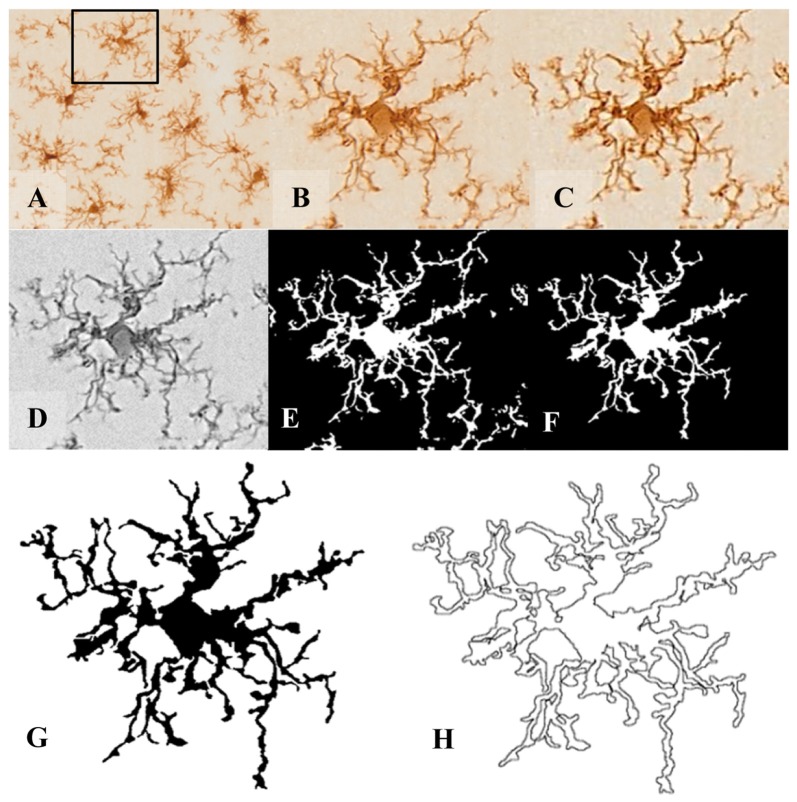
Pre-processing of cell digital image. After random selection of cells from the tissue picture **(A,B)**, the noise was removed by filtering the overall background to get a shape extraction **(C)**. Next, the image was changed to grayscale **(D)**, and then transformed into a binary image **(E)** using the same threshold for all pictures. **(F)** The binary image was edited to clear the background and to join all branches, so that the cell image would be formed by a continuous set of pixels. Finally, a filled shape **(G)** and its pairwise outline shape **(H)** were used for morphological parameters measures.

The morphometric analysis was carried out in DAB-stained microglial cells labeled with IBA-1 antibody (Figures 1A–C). For this purpose, cells were selected and cropped according to the following criteria: (i) random selection starting from the area nearest to the ventricle towards the brain parenchyma up to a depth of about 100 μm; (ii) no overlapping with neighboring cells; and (iii) complete nucleus and branches (at least apparently). Selection was done blinded to treatment. A total of 10 cells per brain area were selected from each animal.

In order to analyze several morphological parameters, each single cell cropped image was processed in a systematic way to obtain a filled image (Figure 1G), and its counterpart outlined shape (Figure 1H). For this purpose, a series of steps were performed using *FIJI* free software (freely downloadable from https://imagej.net/Fiji): (1st) the image was filtered to soften the background and enhance the contrast (Figures 1B,C, respectively); (2nd) it was then transformed to 8-bit grayscale (Figure 1D); and (3rd) binarized to obtain a black and white image by applying a previously established threshold (Figure 1E). (4th) With the aim of obtaining a cell image formed by a single and continuous set of pixels, each image was manually edited: some pixels were cleared to separate ramifications pertaining to neighboring cells, while some pixels were added to join processes belonging to the selected cell. This skilled step was carefully done under the view of the original image of the cell, with special care to avoid bias (compare Figure 1E with 1F). (5th) The filled shape was then cropped (to place the pattern in the center of the picture) and saved to analyze (Figure 1G); and (6th) finally the filled figure was processed to extract and save the outlined shape (Figure 1H).

### Morphometric Analysis

**Figure 2:**
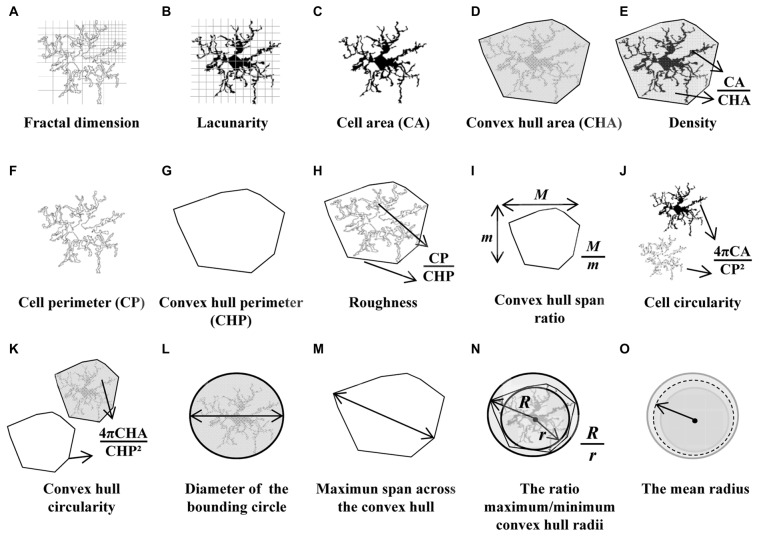
Schematic drawings of the parameters used to analyze the morphology of the microglial cells. *Fractal dimension* **(A)** and *lacunarity* **(B)** were measured by the box counting method. *Cell area* **(C)** and *cell perimeter* **(F)** were assessed by counting cell shape pixels, and both were used to calculate the *cell circularity* (CC) **(J)**. Shape measures such as *convex hull area* (CHA) **(D)** and *convex hull perimeter* **(G)** were necessary to quantify *convex hull circularity (CHC)* **(K)**, *convex hull span ratio* (CHSR) **(I)** and *maximum span across the convex hull* (MSACH) **(M)**. The bounding circle was required to obtain the *diameter of the bounding circle* **(L)**. Finally, some parameters were appraised by combining previous estimations such as *density* **(E)**, *roughness* **(H)**, the* ratio maximum/minimum convex hull radii* **(N)** and the *mean radius* **(O)**.

With the aim of quantifying the morphological changes of microglial cells over the course of inflammation, 15 parameters were measured with the free sofware *FracLac* for* ImageJ* (Karperien, A., FracLac for ImageJ^1^ 1999–2013; available at the ImageJ website, National Institutes of Health; Karperien et al., 2013). Those parameters, measured on the filled and outlined processed images obtained as previously described, were the following (Figure 2):

1. *Fractal dimension* (*D*) is a recognized method to identify intermediate microglial forms ranging from simple rounded to complex branched (Karperien et al., 2013). A higher *D* means a greater complexity of the pattern. Mandelbrot (Mandelbrot, 1983) defined a mathematical approach to describe fractal patterns, where *D* is the exponent to which scale (ε) is raised to get the number of identical parts to itself (*N*_ε_; Equation 1). Therefore, *D* can be calculated from the ratio of ln *N*_ε_ to ln ε within a structure (Equation 2) as the slope of a simple linear regression between ln *N*_ε_ vs. ln ε data.
  (1) $$N_{\varepsilon }\text{= }\varepsilon^{D}$$
  (2) $$D=\text{ln}N_{\varepsilon }\text{/ln}\varepsilon$$
  Box counting software was used to count the number of boxes containing any foreground pixels of the outlined pictures processed along successively smaller caliber grids (Figure 2A). The box size scale was obtained as power series; that is, the base is raised to the exponent added to it to make successive sizes. The slope finally obtained for each image was the average of 12 measurements with different and random placement of the grid.
2. *Lacunarity* (Λ) is associated with changes in the soma and additional morphological features. This parameter measures heterogeneity or translational and rotational invariance in a shape (Karperien et al., 2011). Low Λ value infers homogeneity, having the different parts of an image similar variance. On the contrary, high Λ measurements imply heterogeneity, containing the image many differently sized gaps or lacunas. The Λ calculated with the box counting software *FracLac* is a mass distribution of pixels from microglia images (Figure 2B). The value of Λ obtained was a coefficient of variation expressed as pixel density per box as a function of box size. To avoid bias in Λ results, the mean of mass distribution by power series scales and also with 12 grid locations was calculated (Karperien et al., 2011).
3. *Cell area* was quantified as the total number of pixels present in the filled shape of the cell image (Figure 2C), later transformed to squared micrometers (pixel area = 0.013 μm^2^).
4. *Convex hull area* (CHA), where the convex hull is the smallest convex polygon (that with all interior angles smaller than 180°) containing the whole cell shape (Figure 2D).
5. *Density* was calculated by dividing the *area* of the cell by its CHA (Figure 2E). Some authors call this parameter *solidity*.
6. *Cell perimeter* is measured based on the single outline cell shape (Figure 2F) as the number of pixels expressed in microns (pixel side = 0.115 μm).
7. *Convex hull perimeter* is the single outline of the convex hull (Figure 2G) expressed in microns.
8. *Roughness* was calculated as the ratio of *cell perimeter* to *convex hull perimeter* (Figure 2H).
9. *Convex hull span ratio* is the ratio of the major to the minor axes of the convex hull (Figure 2I). This parameter is also known as *form factor*.
10. *Cell circularity* (CC) was calculated as (4π × *cell area*)/(*cell perimeter*)^2^ (Figure 2J). The circularity value of a circle is 1.
11. *Convex hull circularity* (CHC) was calculated as (4π × *convex hull area*)/(*convex hull perimeter*)^2^ (Figure 2K).
12. *Diameter of the bounding circle* is the diameter (expressed in μm) of the smallest circle that encloses the convex hull (Figure 2L).
13. *Maximum span across the convex hull* (MSACH) is the maximum distance between two points across the convex hull (Figure 2M).
14. *The ratio maximum/minimum convex hull radii* is the division of the largest to the smallest radius from the center of mass of the convex hull to an exterior point (Figure 2N).
15. *The mean radius* was calculated as the mean length in microns from the center of mass of the convex hull to an exterior point (Figure 2O).

### Hierarchical Cluster Analysis

With the aim of identifying similar types of microglial cells, a HCA was performed based on the measured morphometric parameters. SPSS Statistics software, version 21.0 (IBM Corp., Armonk, NY, USA) was used for this purpose. Distance or similarity measures were generated measuring Euclidean distance (the square root of the sum of the squared differences between values for the items) by the Ward’s method (Ward, 1963) for interval data. All data were normalized in order to obtain values in a similar scale. A dendrogram plot based on the Euclidean distance^2^ was used to display the number of potential clusters. To find out the more suitable parameters for separating our population of cells into different cell types, the multimodality index (MMI) of each parameter was calculated (Scheweitzer and Renehan, 1997) using the following formula:

(3) $$\text{MMI}=\left[M{\text{3}}^{2}+\text{1}\right]/[M\text{4}+\text{3 (}n\;-{\text{1)}}^{2}/\text{(}n\;-\text{2) (}n\;-\text{3)}]$$

where *M*3 is skewness, *M*4 is kurtosis, and *n* is the sample size.

The number of appropriate clusters was estimated by the Thorndike procedure (Thorndike, 1953). Briefly, the average within-cluster distance is plotted for different numbers of clusters, resulting in a curve that shows a decrease in the distance as the number of clusters increases. The number of clusters finally selected was revealed by a sudden flattening of the curve in the plot.

Additionally, the Calinski-Harabasz criterion was performed to evaluate the optimal number of clusters; this index is sometimes called the variance ratio criterion (VRC). The optimal number of clusters is the solution with the highest Calinski-Harabasz index value (Caliński and Harabasz, 1974). The VRC is calculated as:

(4) $${\text{VRC}}_{k}=\left(SS_{B}/SS_{W}\right)\times \left[\left(N-k\right)/\left(k-\text{1}\right)\right]$$

where *SS*_B_ is the overall between-cluster variance or sum of squares, *SS*_W_ is the overall within cluster variance or sum of squares, *k* is the number of clusters and *N* is the number observations.

### Linear Discriminant Analysis

The aim of this analysis was to identify characteristics that differentiate the groups and also, to create a function able to distinguish, as accurately as possible, members of each group. The following equation shows linear discriminant functions:

(5) $$LD=A_{1}X_{1}+B_{2}X_{2}+\ldots \;A_{n}X_{n}+C$$

where *A*_n_ is the coefficient of individual morphometric parameters, *X*_n_ is each variable (the morphometric parameter value) and *C* is a constant. Then, a number of discriminant functions equal to *g* − 1 were generated (where *g* is the number of groups being discriminated, that is, four groups or clusters in our case). Some functions may offer a higher discriminant power than others. The *F* statistic value was used to contrast the hypothesis between group means. Additionally, the selection of variables was considered by the global Wilks’s *lambda* value, which was evaluated by the chi-squared transformation. The values of standardized coefficients show the net contribution of each variable to the discriminant function. The function was considered satisfactory when the predictive ability of the discriminate function exceeded 90%. The centroid of each group and the boundaries in the territorial map describe the predicted groups (Fisher, 1936; Yamada and Jinno, 2013; Ohgomori et al., 2016). LDA was carried out by SPSS Statistics software.

### Principal Components Analysis

To assess the overall picture of phenotypic variations a principal components analysis (PCA) was carried out. The two PC should exceed more than 70% of the accumulated variance (Soltys et al., 2005). Distribution of cells values were plotted on the component plane by the SPSS Statistics software. The color code of the cells was based on the hierarchical cluster classification and on the NA/saline treatment. The selected components were supplementary to the main variables suggested by the previous LDA.

### Statistical Analysis

Comparisons of data were carried out using SPSS Statistics software. The Kolmogorov-Smirnov normality test, along with the Levene homoscedasticity test, were used to verify if data could be analyzed by parametric methods. Two-way analysis of variance (ANOVA) was used to compare morphometric parameter values, using “Time” as Factor 1 and “treatment” as Factor 2, with three and two levels, respectively. The Kruskal-Wallis test was performed for the non-parametric data. In the pairwise comparison of morphological parameters the Tuckey test was used. For two sample comparisons, Student’s *t*-test was used for parametric data, and Mann-Whitney *U* test in the case of non-parametric data. Differences were considered significant when a *P* value < 0.05 was obtained.

## Results

### Intracerebroventricular NA Induced Morphological Changes in Microglial Cells in the Septofimbrial Nucleus, the CA3 Hippocampal Area and the Periventricular Hypothalamus

**Figure 3:**
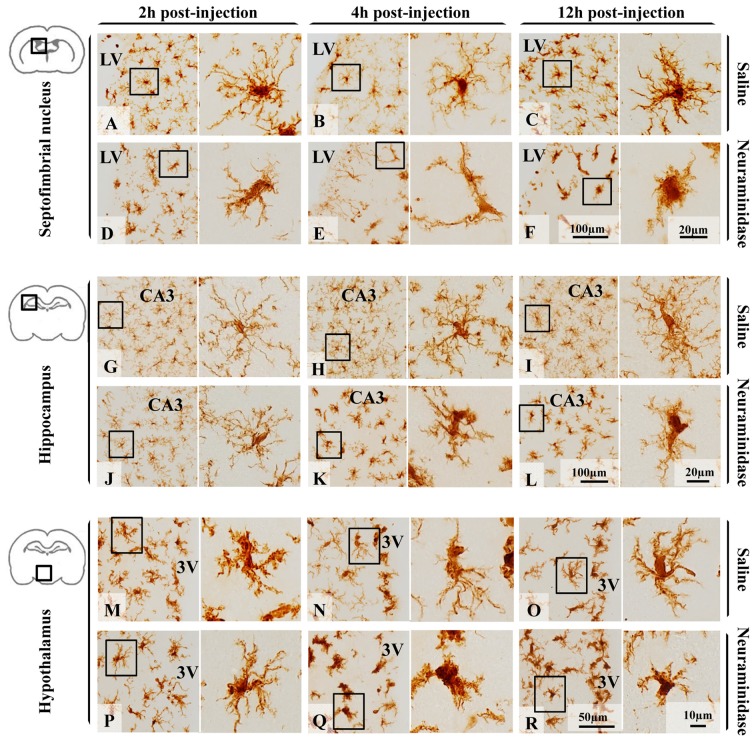
Polymorphism of microglial cells from different brain areas in basal conditions and after an inflammatory stimulus. Microglia morphology, evidenced by the size and form of the soma, and the presence and the length of secondary/tertiary branches, remained unchanged 12 h after saline administration within the LV in the three areas studied: the septofimbrial nucleus **(A–C)**, the hippocampus **(G–I)** and the hypothalamus **(M–O)**. Neuraminidase injection resulted in morphological changes of microglial cells in the septofimbrial nucleus **(D–F)**,the hippocampus **(J–L)** and the hypothalamus **(P–R)**. Only 2 h after neuraminidase (NA) injection, cells in the septofimbrial nucleus showed a larger soma **(D)**, and thicker primary branches after 12 h **(F)** compared to the pairwise sham samples **(A–C)**. In basal conditions microglia located in the hippocampus **(G–I)** exhibited a higher degree of ramification compared to cells in other areas **(A–C,M–O)**. These profuse ramifications appeared slightly decreased 2 h after NA injection **(J)**, and clearly reduced 4 h **(K)** and 12 h **(L)** later. Microglia in the hypothalamus presented a different shape to that located in the hippocampus or septofimbria, with low homogeneity, a large soma and thick branches that could be observed in samples from saline treated rats **(M–O)**. A significant drop in branch length was observed 4 h **(Q)** and 12 h **(R)** after NA injection. LV, lateral ventricle; CA3, field CA3 of the hippocampus; 3V, third ventricle.

Few hours after NA injection, morphological changes of microglia were observed in different brain areas (Figure 3) nearby the ventricular wall, as the injection is performed within the right LV. For the present study, three regions located close to the ventricular surface were selected (the septofimbrial nucleus, the CA3 hippocampal area, and the periventricular hypothalamus) based on the evident morphological change of microglia (and later confirmed activation by IL-1β staining), and because they are relevant structures in cognitive processes and for neuroendocrine integration. At first sight, microglial cells labeled with IBA-1 ranged from ramified in saline treated animals (Figures 3A–C,G–I,M–O) to de-ramifying or bushy forms in NA injected ones (Figures 3D–F,J–L,P–R). However, microglia polymorphism was also evident within the control group treated with saline. Thus, cells from hippocampus (Figures 3G–I) were highly branched with thin extensions and small round or oval somas, while those in the septofimbrial nucleus (Figures 3A–C) displayed less secondary processes and a larger cell body. Furthermore, cells in the hypothalamus (Figures 3M–O) appeared hypertrophied, with shorter and thicker branches, a morphology similar to that observed in NA treated animals (Figures 3D,K,L). Bushy microglia, with short and poorly ramified processes of different thickness around swollen cell bodies, were found in several locations of NA treated samples (Figures 3F,Q,R). Occasionally, some cells showed a highly heterogeneous form (Figure 3E).

### IL-1β Expression Evidences an Acute Neuroinflammatory Reaction Provoked by NA in Periventricular Areas

**Figure 4:**
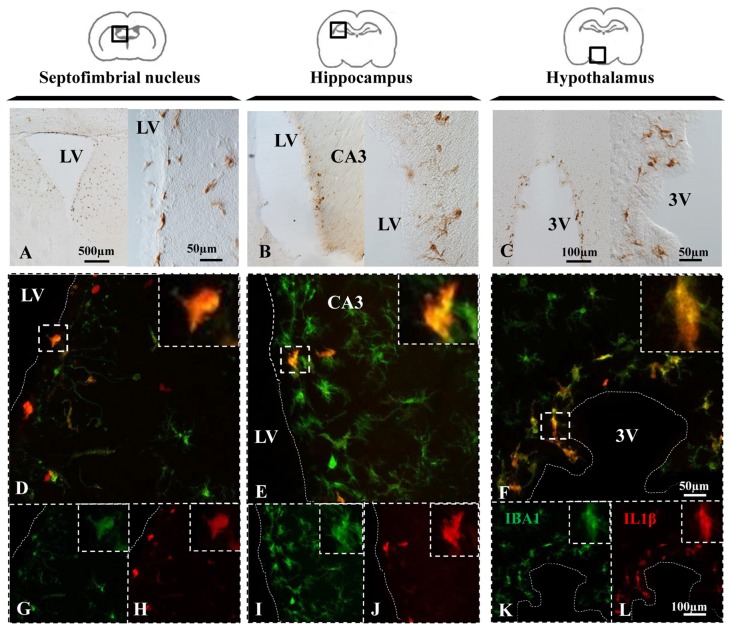
NA induces IL-1β expression in microglial cells. **(A–C)** Brain sections of rats sacrificed 12 h after NA injection were immunostained for IL-1β. Positive cells appeared near the ventricle in all the areas studied, indicating an inflammatory reaction. **(D–L)** Parallel sections from the septofimbrial nucleus **(D,G,H)**, hippocampus **(E,I,J)** and hypothalamus **(F,K,L)** were double-labeled by immunofluorescence with IBA1 (green) and IL-1β (red) antibodies. Some cells show label with both makers, while many others do not express IL-1β. Samples from saline injected animals did not display IL-1β staining (data not shown). Dashed lines indicate the ventricular surface. LV, lateral ventricle; CA3, field CA3 of the hippocampus; 3V, third ventricle.

Upon appropriate stimulation, resident microglia and macrophages infiltrated from systemic circulation become polarized towards a pro-inflammatory M1 phenotype, which is characterized by the synthesis of cytokines such as IL-1β (Orihuela et al., 2016). In NA injected animals, immunohistochemistry revealed IL-1β positive cells in the periventricular areas of the septofimbrial nucleus (Figure 4A), the CA3 of hippocampus (Figure 4B), and the hypothalamus (Figure 4C) 12 h after the injection. In some NA treated animals, IL-1β labeling was detectable even at shorter post-injection times (2–4 h), and no label at all was observed in saline injected animals (data no shown). Thus, in this model NA could be considered to produce a sterile inflammatory reaction that drives microglia towards a pro-inflammatory phenotype.

Double immunofluorescence showed a broad colocation of IL-1β in IBA-1 positive cells in all the studied areas, the septofimbrial nucleus (Figure 4D), the hippocampus (Figure 4E) and the hypothalamus (Figure 4F). However, many IBA-1 positive cells did not display IL-1β staining (Figures 4D–F). IL-1β positive cells were mostly located nearby the ventricular surface (cells for this study were sampled within 100 μm from the ventricle).

### Five Quantitative Morphological Parameters of Microglia Were Statistically Different in Cells from NA Treated Animals Compared to Saline Controls

**Figure 5:**
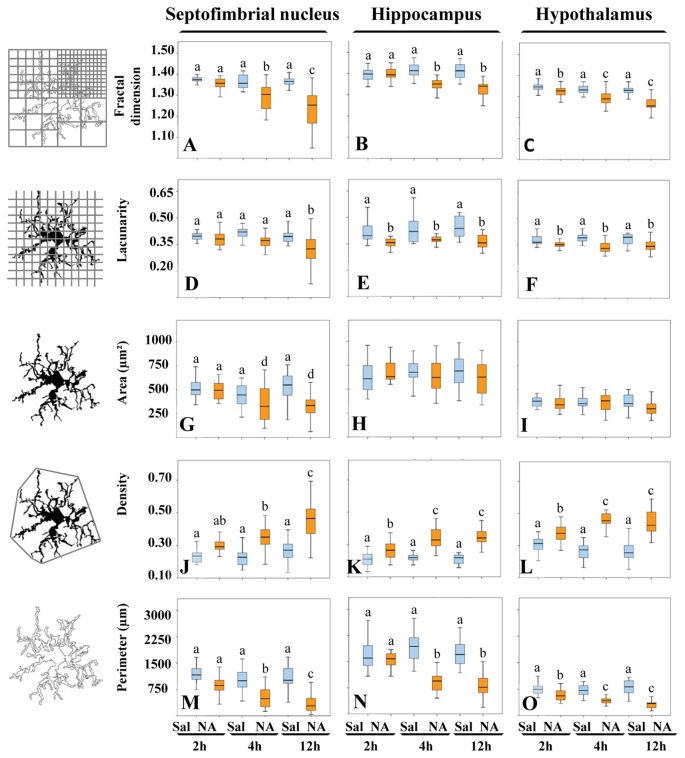
The morphological parameters *fractal dimension*, *lacunarity*, *cell area*, *density* and *cell perimeter* reveal microglial activation after NA treatment. A morphological analysis of microglia from different brain areas was carried out in samples from NA treated and sham rats. Twelve hours after NA injection, all parameters changed significantly compared to saline injected animals, in the three selected areas: the septofimbrial nucleus, the hippocampus and the hypothalamus. However, the parameter *area* did not change in microglia from hippocampus **(H)** and the hypothalamus **(I)**, even though the rest of parameters revealed microglial activation in those regions. Activation of microglia was evidenced by lower *fractal dimension* **(A–C)** and *lacunarity* **(D–F)**, indicating decreased branch complexity and heterogeneity respectively, a higher *density* **(J–L)**, what implies a more compact shape, and a lower cell perimeter **(M–O)**. Pair comparisons results are shown with letters on top of each bar of the histograms. Within each graph, the same letter means no significant difference between the groups, while different letters indicate a statistically significant difference. If no letter appears on top of a bar, no differences exist between any group **(H,I)**. In **(A–F)** and **(J–O)** a, b, c = *P* < 0.001. In **(G)** a, d = *P* < 0.05.

Morphological analysis of a total of 480 microglial cells was performed by measuring five different parameters. *Fractal dimension* (*D*), determined by the box counting method, showed a statistically significant decrease (*P* < 0.001) in NA treated samples with respect to saline ones in the three areas studied (Figures 5A–C), which was more pronounced in the septofimbrial nucleus 12 h after NA administration (Figure 5A). This reduction in *D* values indicate a lower pattern complexity (Karperien et al., 2013), that is, less branch complexity of microglial cells after NA treatment. In saline controls, *D* values about 1.40–1.45 were obtained in the hippocampus (Figure 5B), while those values ranged 1.30–1.35 in the septofimbrial nucleus (Figure 5A) and the hypothalamus (Figure 5C). Those differences in *D* values in basal conditions indicate heterogeneity of microglial populations between different brain areas.

In the case of the parameter *lacunarity* (Λ), a statistically significant decline (*P* < 0.001) was also observed after the administration of NA (Figures 5D–F), with values ranging from 0.41–0.44 in saline samples and 0.31–0.37 in NA treated ones. The decrease was evident as soon as 2 h after the injection in hippocampus (Figure 5E) and hypothalamus (Figure 5F). Since Λ assess heterogeneity or translational and rotational invariance in an image, lower Λ values imply homogeneity (Karperien et al., 2011), i.e., under the influence of NA microglial cells change towards a more homogenous shape.

The parameter* area* was clearly different in microglial cells from different brain regions (compare Figure 5H with 5I), again indicating various microglial populations. However this parameter did not change in hippocampus (Figure 5H) nor in hypothalamus (Figure 5I) upon NA treatment. Conversely, in the septofimbrial nucleus (Figure 5G) NA provoked a significant decrease (*P* < 0.05) in microglia *area* 4 h after its injection (505 ± 70 μm^2^ in saline vs. 350 ± 70 μm^2^ in NA).

When *density* was analyzed, values of this parameter were significantly increased (*P* < 0.001) in all the areas studied when NA was administered, and such increase was evident as early as 2 h post-injection of NA (Figures 5J–L). *Density* values doubled in microglial cells in hippocampus (Figure 5K), hypothalamus (Figure 5L) and septofimbrial nucleus (Figure 5J), with values ranging from 0.21 ± 0.01 in saline microglia in hippocampus, up to 0.46 ± 0.02 in NA microglia in hypothalamus. The increase in *density* shows the tendency of the cells to be more compact after NA injection.

Finally, the *perimeter* of the cells was analyzed as well; it decreased significantly (*P* < 0.001) in the three areas studied when NA was injected (Figures 5M–O). As occurred with *density* and *area*, *perimeter* values were different in basal conditions between the studied areas, the hippocampus presenting the highest values (1770 ± 112 μm; Figure 5N), and the hypothalamus the lowest (330 ± 60 μm; Figure 5O). A decrease in cell *perimeter* is indicative of fewer ramifications.

Pair comparisons of the microglia from different experimental groups within each area studied indicates that three different morphotypes can be distinguished, particularly when *D*, *density* and *perimeter* values are compared (letters *a*, *b* and *c* in each graph of Figure 5). *Area* was not sensitive enough to distinguish these three morphotypes (Figures 5G–I). We next searched for a more comprehensive method to classify our microglia population, regardless the brain region or the experimental treatment.

### Hierarchical Cluster Analysis Allows the Identification of Four Types of Microglial Cells Based on Morphological Parameters

**Figure 6:**
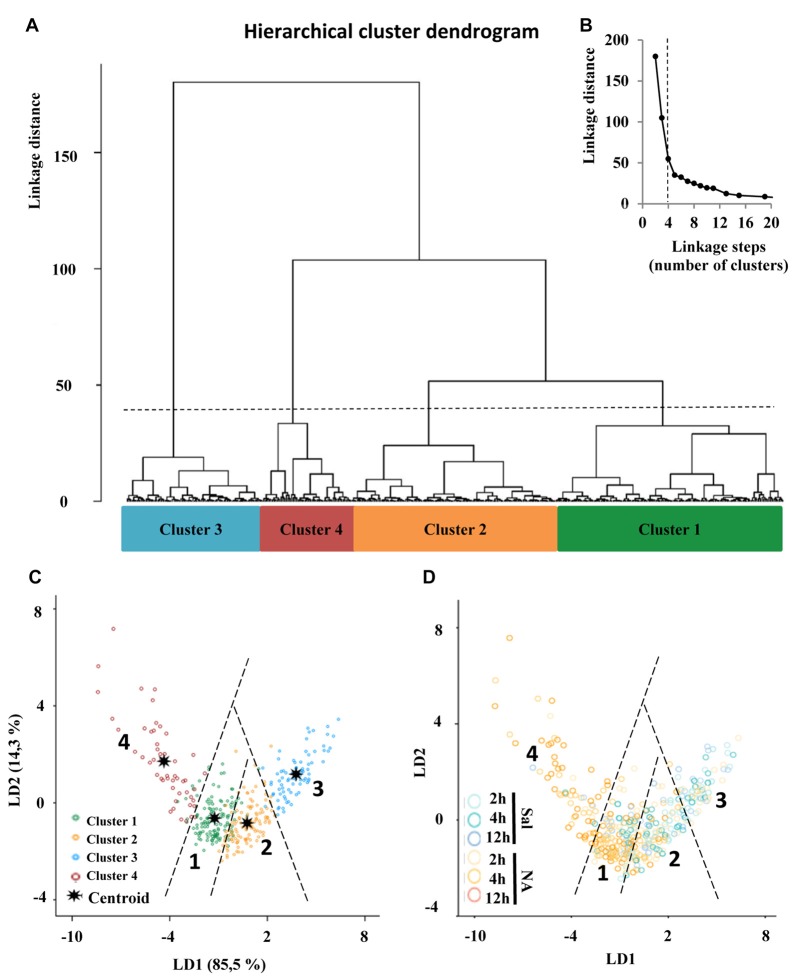
Classification of microglia according to morphological parameters. **(A)** Hierarchical cluster analysis (HCA) of microglial cells sampled from septofimbrial nucleus, hippocampus and hypothalamus of rats after the injection of NA or saline, based on four suitable parameters selected according to their multimodality index (MMI) value. Dendrogram for 480 cells, where the abscissa represents individual microglia and the ordinate corresponds to the linkage distance measured by Euclidean distance. The dashed line denotes the cut off for four clusters, numbered one through four, which were color coded green, orange, blue and dark red, respectively. **(B)** A plot of linkage distance vs. linkage steps (or number of clusters) was performed following Thorndike’s procedure. The vertical dashed line points out a marked decline in the slope, which indicates that four is an appropriate number of clusters. **(C,D)** Territorial mapping of microglial cells on the plane explained by the first two linear discriminant functions (LD1 and LD2); the proportion of trace for each LD is shown in parenthesis. In the graph on the left **(C)** cells are color coded according to cluster allocation; the centroids represent the mean value of each cluster. In the graph on the right **(D)** the same cells are color coded based on saline/NA treatment.

The choice of suitable variables is critical in determining the outcome of HCA. At the first stage of this study, we considered the five parameters explained above, and examined the MMI of those parameters. MMI gives an idea of the distribution of the data around one or multiple values of the parameter. Thus, parameters with MMI > 0.55 are multimodal and therefore suitable to perform cluster analysis (Scheweitzer and Renehan, 1997). Among the previously analyzed parameters, only *cell perimeter* had a MMI greater than 0.55, and the other four had MMI lower than 0.55, so we proceeded to quantify new parameters (up to 15 parameters; Supplementary Material, Tables S2–S4) and calculate their MMI (Table 1). Based on their MMI, the following four morphometric parameters were selected for the cluster analysis: CC, CHA, *cell perimeter* and *convex hull span ratio* (CHSR). The HCA performed on *z*-transformed data sets yielded a dendrogram based on the Euclidean distance between groups, using the Ward’s method (Figure 6A). The Thorndike’s procedure (Thorndike, 1953) was applied to establish the appropriate number of clusters (Figure 6B). This method uses the representation of linkage distance vs. linkage steps (or number of clusters); a sudden decrease in linkage distance occurs at a certain number of clusters, which is evidenced by a marked flattening of the curve. In our case, this happens when the number of linkage steps is four (dashed line in Figure 6B). Microglial cells were thus classified into four clusters (Figure 6A).

**Table 1:** Multimodality index (MMI) of morphometric parameters, and coefficients of linear discriminant functions (LDs) of selected parameters.

Parameter	MMI	LD1	LD2
*Cell circularity*	0.669*	−0.39	0.88^##^
*Convex hull area*	0.577*	0.68^#^	0.63
*Cell perimeter*	0.567*	0.42	0.33
*Convex hull span ratio*	0.558*	−0.68^#^	0.62
*Convex hull circularity*	0.546		
*Max/Min convex hull radii*	0.479		
*Roughness*	0.450		
*Convex hull perimeter*	0.439		
*Density*	0.429		
*Convex hull mean radii*	0.429		
*Cell area*	0.412		
*Diameter of the bounding circle*	0.408		
*Maximun span across the convex hull*	0.404		
*Fractal dimensión*	0.396		
*Lacunarity*	0.393		
Proportion of trace (%)		85.5	14.3

*Selected parameters with MMI > 0.55. ^#,##^ The highest absolute values indicate the best predictor parameters.

Moreover, the VRC values of k-clusters were also determined (Table 2). The higher VRC values point out the most appropriate number of clusters; it appraises both the distance between different clusters, as well as the closeness of data within each of those clusters (Caliński and Harabasz, 1974). In our analysis the VRC value was highest in the case of four clusters (Table 2), thus corroborating the previous cluster definition by the Thorndike procedure. Clusters were numbered according to their size, from the largest to the smallest: Cluster 1, 2, 3 and 4.

**Table 2:** Variance ratio criterion (VRC) following Calinski-Harabasz.

Number of clusters	VRC
2	124.4
3	410.2
4	453.9*
5	347.3
6	410.1
7	451.2
8	446.6
9	124.4

*The highest VRC value indicates the appropriate number of clusters.

### Linear Discriminant Analysis Revealed that *Convex Hull Span Ratio, Cell Circularity* and *Convex Hull Area* Are the Critical Parameters When Sorting Microglial Cells

One of the goals of the LDA is to predict the allocation of a particular microglial cell to one of the four established clusters. Therefore, first we analyzed whether there were significant differences between clusters for each of the independent variables, using group means and ANOVA (Supplementary Material, Table S1). The differences were statistically significant for each variable, suggesting that these variables were good discriminators for microglia classified in the four different clusters.

Next, we searched for linear discriminant functions which could explain the variance, and also suggest the variables that are more relevant for discrimination, i.e., that have the highest predictive capacity. This search resulted in two functions: the linear discriminant function 1 (LD1), with a correlation of 0.929, explained 85.5% of variance, and the linear discriminant function 2 (LD2), with a correlation of 0.716, described 14.3% of variance (Table 1). Thus, both functions together accumulated 99.7% of variance, and only a 0.03% remained unexplained. Moreover, Wilks’s *lambda* and chi-squared pointed out significant differences between the means of the compared groups (Wilks’s *lambda* = 0.479; chi-squared = 275.9; *df* = 6; *P* < 0.001).

The discriminant functions include coefficients for each variable, which are listed in Table 1. The value of those coefficients indicates the partial contribution of each variable to the function, that is, the importance of each variable as predictor of cell sorting in the four clusters, with a higher absolute value indicating a better predictive variable. In the current study the strongest predictors by means of the function LD1 were the *CHSR* and the *CHA*, because they present the highest coefficient values. Regarding the function LD2, the most relevant parameter was *CC* (Table 1).

Besides, the cross validated classification by using LD1 and LD2 functions resulted in 87.9% of the cells correctly allocated in the corresponding cluster.

**Figure 7:**
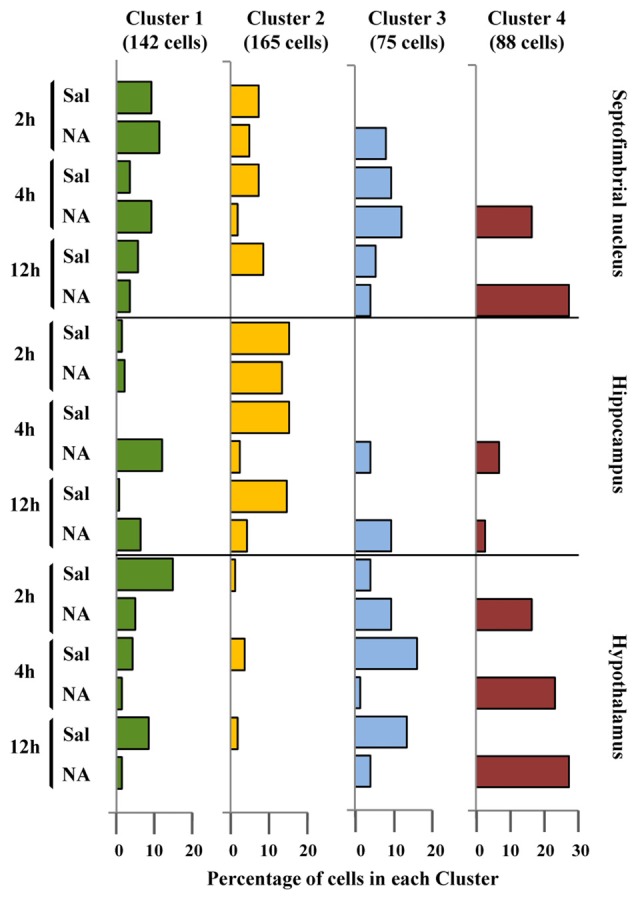
Distribution of microglial cell Clusters in different brain areas. The percentage of microglial cells belonging to the different clusters was plotted considering their brain location, saline/NA treatment and post-injection time. Cluster 1 cells (green bars) slightly decreased after NA injection in the hypothalamus; however in the hippocampus they appeared after NA injection. Cluster 2 cells (yellow bars) were mainly present in saline samples of all brain areas, so they probably represent a surveillant morphotype. Cluster 3 cells (blue bars) were scarce compared to other clusters, and distributed in saline and NA samples. As occurred with Cluster 1, a correlation of Cluster 3 cells with a particular functional state is not easy to establish. Finally, Cluster 4 cells (dark red bars) were exclusively present in NA injected animals; therefore corresponded to activated microglia.

The discriminant scores of 480 microglial cells were determined for LD1 and LD2, and plotted in a territorial map using a color code to identify each cluster (Figure 6C). This type of graph shows how cells within each cluster are grouped around a centroid (the cluster mean). This territorial map was used to see how microglia from NA treated (yellow-orange circles in Figure 6D) and saline control animals (blue circles in Figure 6D) are distributed. Interestingly, most microglia in the territory of Cluster 3 belong to saline controls or NA treated but at short post-injection times, and most cells in Cluster 4 come from NA treated animals. Cluster 1 includes mostly cells from NA treated rats, but also some from saline controls. Cluster 2 includes a mixture of cells from both experimental conditions. This puzzling result could be explained by the following histogram distribution (Figure 7), where the percentage of cells in each cluster was plotted considering the brain area of origin, the saline/NA treatment and the time post-injection. Thus, Cluster 4 clearly represents NA activated microglia in the three brain areas studied, but Cluster 4 cells are more abundant in the hypothalamus (Figure 7). Cluster 1 also includes mostly cells from NA treated animals, but some cells from saline controls are also allocated in this cluster, particularly those from the hypothalamus and the septofimbrial nucleus (Figure 7). Clusters 2 and 3 represent cells in saline controls or cells in NA treated rats but at short post-injection times (i.e., not yet activated), thus clarifying, at least partially, the apparent mixture of phenotypes within these clusters. The histogram shows additional information; for example, resting microglial cells residing in the hippocampus belong mainly to the Cluster 2 (while this phenotype is scarce in the hypothalamus), and the activated phenotypes in this area are distributed between Clusters 3, 4, and preferentially Cluster 1. A further analysis was performed to refine this classification (see next section).

### Principal Components Analysis Revealed that *Maximum Span Across the Convex Hull* and *Convex Hull Circularity* Are Supplementary Features that Aid Microglia Classification

**Figure 8:**
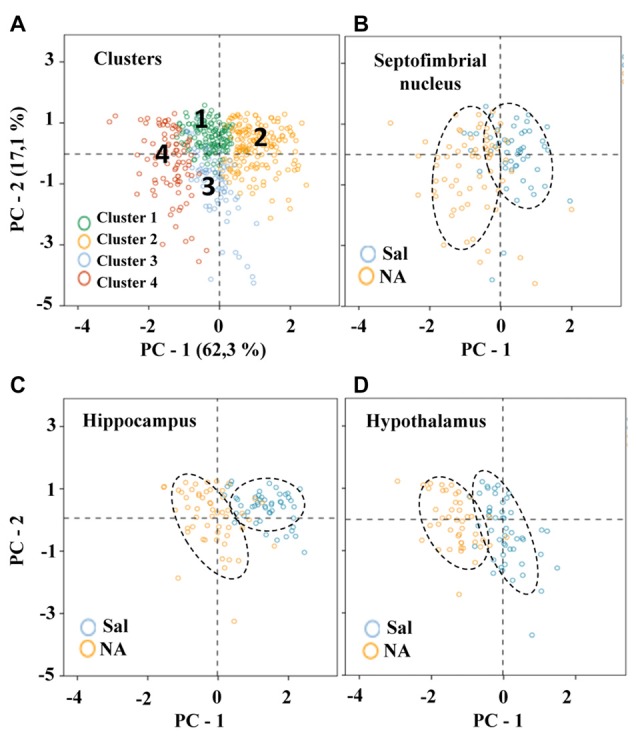
Distribution of microglial cells on the principal components (PC) plane. **(A)** Distribution of microglial cells on the PC plane with projections of the main variables (PC1 and PC2) from coordinates origin, and the percentage of total variance explained by each component. Each cell was color coded according to its Cluster allocation. Cells belonging to the same cluster appear grouped on the component plane. **(B–D)** The same distribution was plotted for each of the studied areas, and using a color code to indicate saline (blue) or NA (yellow) treatment. Dashed elliptical lines were drawn to highlight the region of the plane occupied by each type of cells.

The 11 morphometric parameters describing microglial shape obtained previously were subjected to PCA. As a result, the first two PCs accounted for 79.4% of the observed variability (PC1 = 62.3%, PC2 = 17.1%; Figure 8A; Table 3). In this analysis, some of the 15 parameters can be discarded, because they overlap with others in the same component, PC1 or PC2. Only those parameters with the strongest correlation with each component (i.e., with the highest coefficient values) should be considered (Table 3). Thus, PC1 was highly affected by *convex hull perimeter* or MSACH, while PC2 was mainly influenced by *CHC* or* max/min convex hull radii*. Since the present analysis intended to further classify microglial cells, those parameters with high PC coefficients were chosen. Thus, MSACH was pondered as the main parameter of PC1, and *CHC* for PC2. These parameters were selected based on their potency as discriminators, which was also evaluated by pair comparisons within each of the four Clusters.

**Table 3:** Coefficients of principal components analysis (PCs).

Principal components	PC1	PC2
*Convex hull perimeter*	0.97*	
*Convex hull mean radii*	0.95*	
*Diameter of the bounding circle*	0.91*	0.37
*Maximun span across the convex hull*	0.90*	0.38
*Roughness*	0.89	
*Fractal dimensión*	0.86	
*Cell area*	0.85	
*Density*	−0.72	
*Convex hull circularity*	0.50	−0.80**
*Lacunarity*	0.48	
*Max/Min convex hull radii*	−0.44	0.78
Proportion of trace (%)	62.3	17.1

^*,**^The highest absolute values indicate the parameters with greater influence.

To compare hierarchical cluster results obtained previously with PCA outcome, microglia scores were distributed on the PC plane (Figure 8). Cells were categorized by colors according to their cluster allocation (Figure 8A). Such graph showed that cells belonging to different clusters were grouped in the same region of the PC plane. Thus, the PCA results validate the four cluster partition obtained by HCA. In this way, the PC plane revealed the possibility of separating Cluster 2 and Cluster 4 by the PC2; microglia included in Cluster 2 would present high branch complexity in contrast to cells in Cluster 4, which would exhibit simpler ramifications. On the other hand, PC1 could be used to separate Cluster 1, which would include cells with a more circular shape, from Cluster 3, mostly containing polarized cells (Figure 8A).

**Figure 9:**
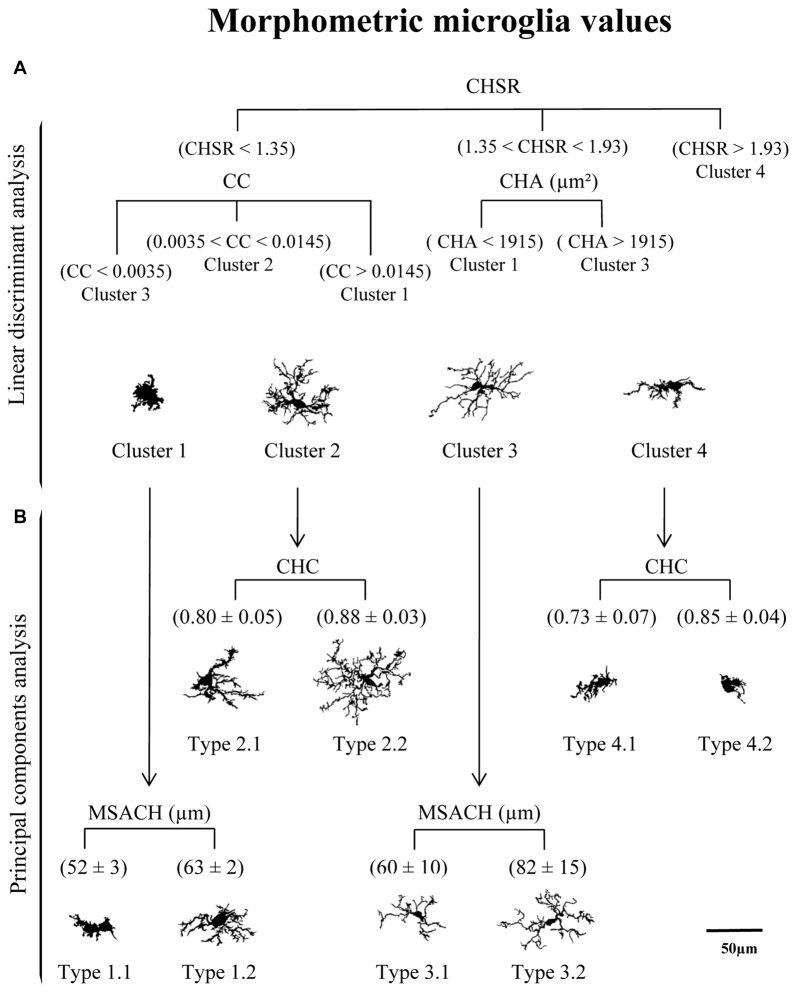
Categorization of microglial cells by means of a logical decision tree based on morphological parameters. **(A)** With the aim of assigning cells into one of the four proposed clusters a logical decision tree was designed based on the strongest predictor parameters revealed by the linear discriminant analysis (LDA). For each individual microglial cell, the first parameter to be analyzed would be the CHSR. Cells with a value greater than 1.93 could be assigned to Cluster 4, while those with lower values would be then evaluated by its CC or its CHA. These two parameters would allow to allocate cells to Clusters 1, 2 and 3, according to the parameter values indicated in **(A)**. **(B)** Principal components analysis (PCA) suggested a further microglia classification with a descriptive categorization. In this case cells within the different clusters were classified based on values respect to the plot axis in the PC plane. The main parameter of PC-1, *MSACH*, allowed to split Clusters 1 and 3, resulting in Types 1.1 and 1.3, and Types 3.1 and 3.2, respectively. In both cases, the resulting Types were significantly different according to their MSACH values (*P* < 0.001). The main parameter of PC-2, *CHC*, was used to split Clusters 2 and 4 in Types 2.1 and 2.2 and Types 4.1 and 4.2, respectively. Differences in CHC between these Types were significant (*P* < 0.001). Cells are all in the same scale (scale bar = 50 μm).

The distribution of cells in the PC plane allows going one step further, since some clusters can be broken down by the coordinate axis. Thus, the values obtained from the main PC parameters were useful to split each of the four clusters into sub-clusters (here called Types; Figure 9B). In this way, Clusters 1 and 3 were subdivided by considering the parameter *MSACH* (selected as major component of PC1 based on its high coefficient value), and Clusters 2 and 4 were further divided by using the parameter *CHC* (selected as a main parameter of PC2). To find out if the pairwise fragmented Types were actually different, the statistical comparison of the means of the discerning parameter was performed. The results showed significant differences (*P* < 0.001) in the parameter MSACH (Figure 9B) between the two new morphotypes (Type 1.1 and Type 1.2) obtained from Cluster 1, as well as between those obtained from Cluster 3 (Type 3.1 and Type 3.2). Similarly, the two new microglial morphotypes obtained according to the parameter CHC (Figure 9B) from Cluster 2 (Types 2.1 and 2.2) and from Cluster 4 (Types 4.1 and 4.2) were also statistically different (*P* < 0.001).

The classification of microglia by LDA was connected to the cell classification performed by PCA, resulting in a tree (Figure 9), where the top panel corresponds to the LDA and has a predictive capacity, and the bottom panel corresponds to the PCA, which is not predictive. In this way, cells were first allotted to four Clusters according to three parameters (CHSR, CC and CHA; Figure 9, top), and later subdivided in eight Types based in two additional parameters (CHC and MSACH; Figure 9, bottom). The range of values of each of the five parameters considered for the decision tree was quite ample, and therefore cells with rather different morphological features may be included (Figure 11).

**Figure 10:**
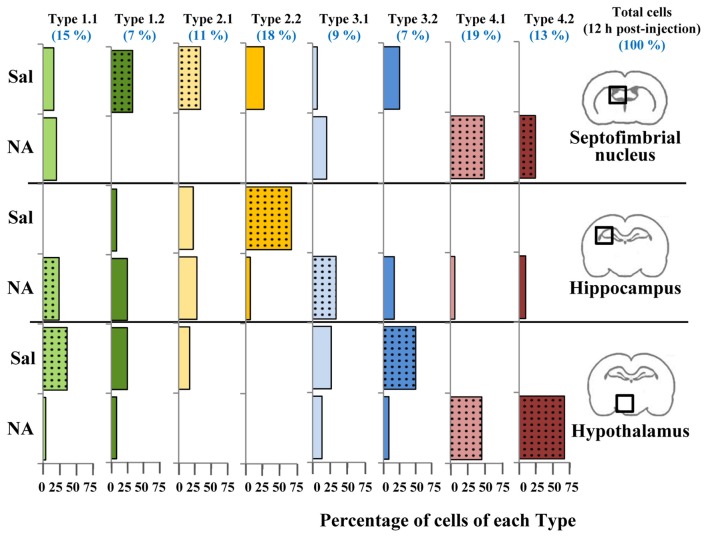
Distribution of microglial cell Types in different brain areas. Microglial cells from 12 h post-injection animals were classified using the previously proposed decision tree. For each cell Type, the distribution (percentage) of cells in different brain areas (septofimbrial nucleus, hippocampus and hypothalamus) and treatments (saline/NA) was plotted as histogram. Color of bars indicate Cluster origin (same color code as Figure 7), using light color for Type x.1 and dark for Type x.2. At the top in blue, the distribution (percentage) in Types of the total cells analyzed (about 150 cells) is indicated. Dotted bars indicate the most representative microglial Type for each brain region and treatment.

**Figure 11:**
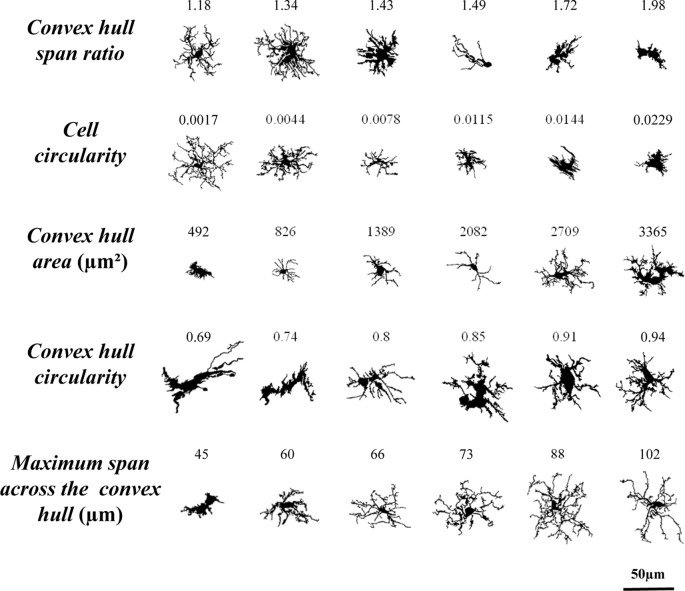
Different microglia filled shapes and their corresponding parameters values. Values of the strongest predictor parameters of LDA and PCA, corresponding to a survey of microglial cells from different brain areas and saline/NA treatments. Parameters values are at the top of each cell. Cells are shown in the same scale (scale bar = 50 μm).

To find out if the four Clusters and the defined eight Types of microglia were related to their different activation states, the scores of microglial cells obtained by PCA were scattered on the PC plane, considering the saline/NA treatment, and the brain area sampled: the septofimbrial nucleus (Figure 8B), the hippocampus (Figure 8C), and the hypothalamus (Figure 8D). In each brain area, cells from saline or NA treatments were grouped in the same region of the plane (dashed ovals in Figures 8B–D). However, these regions did not completely overlap when comparing different brain areas (compare saline in hippocampus Figure 8C, with saline in hypothalamus Figure 8D), suggesting that microglial cells residing in those areas are morphologically distinct, and therefore would be classified as different Clusters or Types. Furthermore, the resting morphology of cells in hypothalamus (blue circles in Figure 8D) widely overlaps with NA activated cells in hippocampus (orange circles in Figure 8C), indicating that the resting vs. activated morphology is dependent on the brain area.

**Figure 12:**
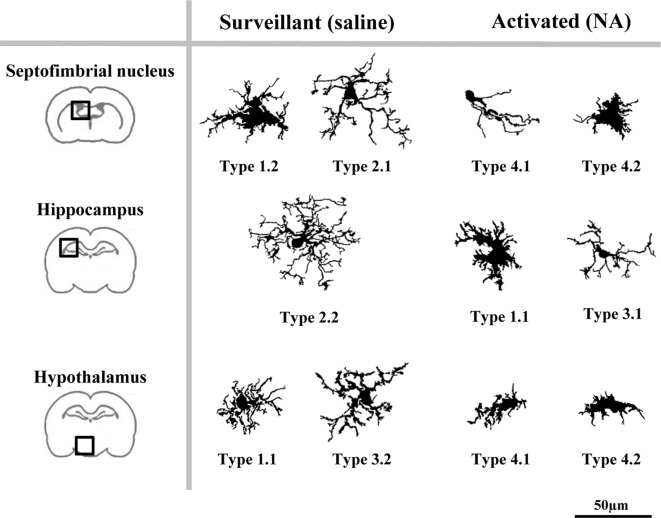
A proposed model of the distribution of microglial morphotypes in the rat brain subjected to induced aseptic inflammation. Surveillant microglia are represented by morphotypes of Cluster 1 (Types 1.1 and 1.2) and Cluster 2 (Types 2.1 and 2.2). An additional surveillant morphotype (Type 3.2) can be found in the hypothalamus. Type 2.2 is almost exclusive of the hippocampus, and is the main surveillant form found in this brain region. Upon activation by NA injection, the most representative morphotypes are Types 4.1 and 4.2 derived from Cluster 4, and Type 3.1. However, morphotypes derived from Cluster 1 can also be found in brains treated with NA (e.g., Type 1.1 in hippocampus). Therefore, some Types are clearly associated to surveillant (2.1, 2.2, 3.2) or activated (3.1, 4.1, 4.2) forms of microglia, while others (Type 1.1) can correspond to either state. For this reason, to define the activation status of a particular microglial cell its brain location must be taken into account. Cells are shown in the same scale (scale bar = 50 μm).

As a result of the decision tree, a new histogram was obtained, presenting the percentage of cells of each Type within each brain region (Figure 10). For simplification, only cells from 12 h post-injection samples were included. According to this histogram, Type 2.1 mostly represents surveillant microglia from septofimbria, while Type 2.2 represents surveillant microglia in hippocampus. This does not mean that these phenotypes are not present in other regions, but here we emphasize the most typical location of each Type. This histogram also shows that Cluster 4, as well as the subtypes derived from it, include activated microglia. In this case, Type 4.1 and Type 4.2 are found both in septofimbria and in hypothalamus; while Type 4.1 is more abundant in septofimbria, Type 4.2 is more frequent in hypothalamus. As previously exposed, Cluster 1 and Cluster 3 include a mixture of surveillant (saline) and activated (NA) phenotypes. However, the histogram helps to clarify this issue. Type 1.1 is a surveillant phenotype in hypothalamus, but an activated phenotype in hipoccampus, while Type 1.2 a surveillant phenotype in septofimbria. Therefore, the same Type (1.1) may represent different activation phenotypes depending on the brain region. Similarly, Cluster 3 cells distribute between surveillant and activated cells; Type 3.1 represents an activated state in the hippocampus, while Type 3.2 represents a surveillant phenotype in hypothalamus. In this way, the distribution of microglial cells from each Type in the different brain areas and under different activation states is settled (Figure 12). However, as the histogram shows, this is evaluated in terms of abundance, as most Types are not exclusive of a region or treatment (saline/NA).

## Discussion

### Morphometric Parameters Reveal the Activation of Microglia Induced by NA

Our experimental model consists in the induction of a neuroinflammatory process by injecting NA within the LV of rats (Grondona et al., 1996; Del Carmen Gómez-Roldán et al., 2008). This aseptic inflammation affects areas nearby the ventricular wall in different brain structures, such as the septofimbrial nucleus, the hippocampus and the hypothalamus. Twelve hours after NA injection, most microglial cells exhibit IL-1β immunostaining (Figure 4), indicating that they are polarized to a pro-inflammatory M1 phenotype (Olah et al., 2011; Walker et al., 2014; Orihuela et al., 2016). But we also find IL-1β negative microglial cells, which can be considered as surveillant, in saline controls or NA treated samples but at short post-injection times. Thus, we are dealing with a microglia population in diverse activation states.

With the aim of classifying microglial cells according to their morphology and activation status, five morphological parameters were measured for each sampled cell: *fractal dimension* (*D*), *lacunarity* (Λ), *area*, *density* and *perimeter*. In the three brain areas selected for this study, all these parameters were statistically different in microglial cells from NA treated animals, comparing with saline controls or even with NA treated rats but at short post-injection times (Figure 5). Thus, morphological changes can be tightly linked to the conversion of microglia from a surveillant state to an activated state, i.e., to the polarization to M1 phenotype.

Comparing with reports by other authors, the *D* values obtained here (within a range of 1.42–1.23) agree with the presence of the four widely described forms of microglia: (i) ramified resting/inactive; (ii) hypertrophied; (iii) bushy; and (iv) unramified reactive/amoeboid/activated (Fernández and Jelinek, 2001; Karperien et al., 2013). After NA injection, *D* values of microglial cells decreased in the three areas studied (Figures 5A–C), as described (Fernández and Jelinek, 2001; Karperien et al., 2013). However, other studies found an increase in this parameter after brain injury, schizophrenia, Alzheimer’s disease or affective disorders (Soltys et al., 2001; Karperien et al., 2008). *Lacunarity* (Λ) values also decreased after NA treatment (Figures 5D–F), indicating a change of cells shape to a more homogeneous morphology (Karperien et al., 2011). Microglia classification based on *D* results is complemented by Λ data; a strong proportional correlation between *D* and Λ has been described in reactive microglia (Karperien et al., 2011), what is in accordance with our results.

Regarding other morphometric parameters, some studies reported substantial variability in microglial cells from healthy brain cortex (Kongsui et al., 2014; Torres-Platas et al., 2014), and phagocytic microglia (Perez-Pouchoulen et al., 2015), and also in microglia present in pathological situations such as induced chronic stress (Hinwood et al., 2013) or ischemic stroke (Morrison and Filosa, 2013). For example, microglia *perimeter* decreased after systemic lipopolysaccharide (LPS; Kozlowski and Weimer, 2012; Kongsui et al., 2015), permanent occlusion of the middle cerebral artery, or traumatic brain injury (Zanier et al., 2015). Our *perimeter* results agree with these; the injection of NA induced a reduction in microglial cells *perimeter* (Figures 5M–O), which is in accordance with the identification of different morphotypes based on *perimeter* values. In our model, the parameter *density* (also called *solidity*) increased after NA treatment (Figures 5J–L). In all areas such increase was coherent with the presence of different microglia morphotypes based on *density* values. Other authors also reported increases in *density* values after brain injury (Soltys et al., 2001; Zanier et al., 2015) or LPS injection (Kongsui et al., 2015). However, other studies showed an opposite tendency with an increase in microglia *perimeter* after temporal occlusion of middle cerebral artery (Zanier et al., 2015). Finally, changes of the parameter *area* in our inflammation model were not consistent, as we observed a decrease after NA injection but only in one of the brain areas studied (the septofimbrial nucleus; Figure 5G). A similar resistance to change of this morphometric parameter in microglial cells has been described in mouse models of Alzheimer’s disease (Baron et al., 2014), in microglia activated by ageing, or surgically injured rats (Hovens et al., 2014). Therefore, we can deduce that, at least in our model, *fractal dimension*, *lacunarity*, *density* and *perimeter*, but not cell *area*, are sensitive morphologic indicators of microglial cell activation.

Our microglia morphometric results fit with certain categorical morphotypes previously described (Fernández and Jelinek, 2001; Karperien et al., 2013). First, a ramified surveillant phenotype, with small cell body, long primary branches and quite thin secondary branches, would be present in saline injected animals, and would be described by high *D*, Λ and *perimeter*, and a low *density*. Four hours after NA injection, another morphology would start to show up, which could correspond to de-ramified microglia, with larger soma and shorter and thicker processes; this microglia morphology would be described by lower *D*, Λ and *perimeter* values, and increased *density*. Finally, a third morphotype, present in samples 12 h after NA injection, would be unramified reactive/activated microglia with large swollen body alongside few thick branches; it would be characterized by the lowest *D*, Λ and *perimeter* values, and the highest *densities*. This latter morphotype could also be associated to the M1 polarized.

### Hierarchical Cluster and Principal Components Analysis Allow the Morphological Classification of Microglia

In order to properly categorize the different morphotypes of microglia present in our experimental model and correlate them with their activation state, we performed a HCA (Figure 6A). In this mathematical approach, the choice of the parameters is crucial in determining the outcome of the analysis; multimodal datasets are the most appropriate for separating a population of cells into morphotypes (Scheweitzer and Renehan, 1997). In this sense, only some authors consider the MMI to make their parameter selection. This more rigorous approach rendered two microglia morphotypes after Dengue infection (Diniz et al., 2016), and four types of reactive microglia following hypoglossal axotomy (Yamada and Jinno, 2013). In our neuroinflammatory model, we measured 15 morphometric parameters and analyzed their datasets by MMI. Although we had previously found statistical differences in *D*, Λ and *density* when comparing NA treated with control (saline) microglia, the MMI for those parameters turned out to be not suitable for cluster analysis. Instead, *CC*, *CHA*, *cell perimeter* and *CHSR* presented MMI values greater than 0.55, and were therefore chosen for cluster classification of microglia (Table 1). Based on these parameters, and following Thorndike’s procedure (Thorndike, 1953), microglia were classified in four clusters or morphotypes (Figure 6B). The distribution of microglial cells on a territorial map by LDA (Figure 6C) showed that Clusters 3 and 4 were the most distant, and therefore they probably correspond to the most different morphotypes in our model. Clusters 1 and 2 were closer to each other. When scattering microglial cells in the same territorial map but using a color code related to saline/NA treatment (Figure 6D) Cluster 3 overlaps with microglia from saline samples, that is surveillant, and Cluster 4 concur with microglia from NA treated animals, namely activated microglia. Clusters 1 and 2 included a mixture of cells from saline and NA samples. Therefore, although Clusters 1 and 2 represent two different microglial populations according to their morphology, they are not suitable for a proper classification of microglia as surveillant or activated. Based on this result, although a correlation between form and activation in microglial cells is widely accepted, the definition of the activation state based on morphology is not always straightforward.

To clarify this issue, a PCA was carried out (Figure 8). The PCA considers another set of variables (which may be different to those considered in LDA); after scrutinizing all the morphometric parameters, PCA reveals those with more weight in the variance. After PCA, the selected valuable parameters were *MSACH* and *CHC* (Figure 9). Cells were scattered on a PCA plane using a four color code to indicate their cluster allocation (Figure 8A), or a two color code to note their NA/saline origin (Figures 8B–D). Thus microglia can be displayed from a different point of view to that of LDA. Besides, each cluster cell counts per brain area and type of treatment was represented in a histogram (Figure 7). These additional analyses (PCA and histogram) provided a clearer picture of the morphotypes found in our model. Although LDA pointed Cluster 3 as surveillant microglia, Cluster 2 fits better with the surveillant state, according to its population decrease in all brain areas after NA injection. Cluster 2 morphotype is more abundant in hippocampus and septofimbrial nucleus, but quite scarce in the hypothalamus. Cluster 3 morphotype can be considered as intermediate, as is similarly present in samples from saline or NA treated animals. When considering the area studied, the number of cell in Cluster 3 decrease after NA injection in the hypothalamus, but they increase in the hippocampus. A similar contradiction occurs with Cluster 1, with an increase of this morphotype after NA injection in the hippocampus, and a decrease in the hypothalamus. Finally, Cluster 4 can be unequivocally considered an activated morphotype, as it appears only after NA injection in all areas studies. Therefore, we have developed a useful tool, based on cell morphology, for the classification of microglial cells in different activation states. However it seems quite relevant to take into account the area under study, since results from one area may not correctly be extrapolated to other brain areas. That is, the same microglia morphotype can represent a surveillant cell in a brain region, while being an activated form in another.

If it is complicated to establish a correlation between cluster allocation and activation state in our experimental model, attempting this task comparing our results with those by other investigators is probably unfeasible. Nonetheless, we may relate our Cluster 2 morphotype with the surveillant microglia, always present prior to injury, which has been classified as *ramified microglia* before brain injury (Soltys et al., 2001), as *Type I microglia* before hyppoglossal axotomy (Yamada and Jinno, 2013), or as *Type S microglia* in controls of an ALS mouse model (Ohgomori et al., 2016). On the other hand, our Cluster 4 cells may correspond to an activated morphotype, found after diverse brain experimental/pathological situations, designated as *bushy microglia* in brain injury (Soltys et al., 2001), as *Type IV microglia* after axotomy (Yamada and Jinno, 2013), or as *R3 microglia* in advanced ALS processes (Ohgomori et al., 2016).

### A Proposed Decision Tree Based on Morphological Parameters for Microglia Categorization

After linear discriminant predictive classification of cells into clusters, the scattering of microglia on a PC plane showed that the different clusters were clearly separated, which validated the HCA and LDA classification. However: (i) microglial cells scores were spread along of PC axis; and (ii) Clusters 1 and 3 morphotypes did not correlate properly with an activation state. Therefore, we searched for another subdivision of those clusters. A logical decision tree was proposed (Figure 9), based on the strongest predictive parameters revealed by the LDA, which initially allow allocating cells in the defined clusters, and later splits each cluster based on additional parameters selected according to the PCA. This latter proposed subdivision cannot be considered as predictive as the previous one, since PCA cannot cross validate the suggested classification. However, we considered that PCA could exploit the quantitative description of microglia shape by other parameters, even when the data distribution of those shape features was unimodal. Thus, the first step of the proposed procedure consists in assessing the *CHSR*, the *CC*, and the *CHA* of each microglial cell. These parameters allow the allocation of each cell in one of the four clusters established by LDA (Figure 9A). Then, PCA revealed additional features, namely *MSACH* and* CHC*, can be used (at least in our model) to further categorize cells into a total of eight Types (Figure 9B). The subdivision proposed by PCA should be considered only when the microglia values of principal parameters are clearly polarized; in any case, the differences between the resulting subgroups must be corroborated by a statistical analysis.

The implementation of the aforementioned decision tree (Figure 9) with the help of the histogram distribution of the eight Types within the different brain regions (Figure 10), resulted in certain unambiguous surveillant (Types 2.1 and 2.2) and activated (Types 4.1 and 4.2) morphotypes. Also, it settles the conflicting situation of Cluster 1 and Cluster 3, which include both surveillant phenotypes (Types 1.1, 1.2 and 3.2) and activated phenotypes (Types 1.1 and 3.1) depending on the brain region where microglia is located. The most representative microglial Types in each brain region are summarized in Figure 12. Note that: (1) in most cases, there is not an exclusive cell Type for any brain region or activation state; (2) each morphotype may correspond to surveillant microglia in certain brain areas, and activated microglia in other locations. Therefore, region localization of microglia has to be always taken into account.

In conclusion, this work demonstrates that microglial cells under inflammatory conditions (NA) present statistically different morphological parameters compared to microglia from a control (saline) situation. Four different clusters of microglia were proposed by HCA. Then, LDA suggested three relevant parameters to classify any microglia according to morphological measurements by means of a decision tree. Subsequently, two additional valuable parameters were identified by PCA, which allowed the subdivision of clusters, obtaining a total of eight Types of microglial cells. The allocation of the cells found in our saline/NA model in the proposed Clusters/Types resulted in the dispersed distribution of surveillant microglia (from saline samples) among the proposed morphotypes, while the allocation of activated microglia (from NA samples) was more homogenous (mostly in Cluster 4). Curiously, some surveillant microglia from specific brain areas occasionally showed the same morphotype as activated microglia located in other brain areas, indicating that the surveillant phenotype may be, to some extent, region specific. We suggest that brain location should be considered for future microglia classifications. Therefore, this work establishes a correlation between different morphotypes and activation states of microglial cells, considering as well their brain location. Also, a reappraisal of morphometric parameters by PCA has been proposed, particularly in the case of those parameters with unimodal distribution.

## Author Contributions

MDL-Á, PF-L and MMF-A conceived and designed the study. MMF-Á and PG-D performed the experiments. MDL-Á, JMG and MMF-A analyzed the data. MDL-Á, JMG and MF-A wrote the manuscript. All authors read and approved the final manuscript.

## Conflict of Interest Statement

The authors declare that the research was conducted in the absence of any commercial or financial relationships that could be construed as a potential conflict of interest.

## Footnotes

^1^http://rsb.info.nih.gov/ij/plugins/fraclac/FLHelp/Introduction.htm

^2^http://www.r-project.org
