# Supplementary material for: Microglia Morphological Categorization in a Rat Model of Neuroinflammation by Hierarchical Cluster and Principal Components Analysis
Source: Front Cell Neurosci. 2017 Aug 8;11:235. doi: 10.3389/fncel.2017.00235 (PMC5550745; doi:10.3389/fncel.2017.00235)
Supplement: Supplementary file 1 [file Table_1.docx]

**TABLE S.1.** ANOVA of the parameters used for LDA for clusters comparison.

| **Parameter** | **Wilks´s *lambda*** | ***F*** | **df1** | **df2** | **P** |
| --- | --- | --- | --- | --- | --- |
| *Cell perimeter* | 0.500 | 125.35 | 3 | 476 | < 0.001 |
| *Cell circularity* | 0.477 | 137.51 | 3 | 476 | < 0.001 |
| *Convex hull area* (µm) | 0.242 | 393.44 | 3 | 476 | < 0.001 |
| *Convex hull span ratio* | 0.520 | 115.55 | 3 | 476 | < 0.001 |

**TABLE S.2.** Morphometric values of microglial cells from the septofimbrial nuclei (mean ± SD, n=160)

|  | **2 h** | | **4 h** | | **12 h** | |
| --- | --- | --- | --- | --- | --- | --- |
| **Parameter** | **Saline** | **NA** | **Saline** | **NA** | **Saline** | **NA** |
| *Cell circularity* | 0.005 ± 0.001 | 0.009 ± 0.004 | 0.007 ± 0.006 | 0.022 ± 0.021 | 0.006 ± 0.003 | 0.031 ± 0.020 |
| *Convex hull area* (µm^2^) | 2214 ± 525 | 1695 ± 461 | 1940 ± 770 | 1183 ± 683 | 2135 ± 674 | 886 ± 443 |
| *Cell perimeter* (µm) | 1181 ± 254 | 902 ± 222 | 1040 ± 400 | 592 ± 325 | 1132 ± 374 | 449 ± 223 |
| *Convex hull span ratio* | 1.3 ± 0.2 | 1.6 ± 0.6 | 1.6 ± 0.5 | 1.7 ± 0.7 | 1.4 ± 0.4 | 1.8 ± 0.6 |
| *Convex hull circularity* | 0.86 ± 0.03 | 0.81 ± 0.11 | 0.83 ± 0.07 | 0.77 ± 0.11 | 0.85 ± 0.07 | 0.78 ± 0.10 |
| *Max/Min convex hull radii* | 1.6 ± 0.2 | 1.8 ± 0.5 | 1.9 ± 0.6 | 2.1 ± 0.9 | 1.7 ± 0.4 | 2.0 ± 0.6 |
| *Roughness* | 6.6 ± 0.9 | 5.6 ± 1.1 | 5.9 ± 1.4 | 4.2 ± 1.5 | 6.3 ± 1.4 | 3.7 ± 1.2 |
| *Convex hull perimeter* (µm) | 179 ± 22 | 162 ± 22 | 168 ± 33 | 133 ± 36 | 176 ± 28 | 115 ± 27 |
| *Density* | 0.23 ± 0.04 | 0.32 ± 0.07 | 0.25 ± 0.08 | 0.35 ± 0.13 | 0.27 ± 0.06 | 0.42 ± 0.13 |
| *Convex hull mean radii* (µm) | 29 ± 4 | 27 ± 4 | 28 ± 5 | 23 ± 6 | 29 ± 5 | 20 ± 4 |
| *Cell area* (µm^2^) | 504 ± 108 | 523 ± 123 | 435 ± 118 | 371 ± 195 | 562 ± 193 | 344 ± 139 |
| *Diameter of the bounding circle* (µm) | 66 ± 9 | 63 ± 12 | 64 ± 13 | 52 ± 14 | 66 ± 10 | 45 ± 10 |
| *Maximun span across the convex hull* (µm) | 65 ± 9 | 62 ± 12 | 63 ± 13 | 52 ± 14 | 65 ± 10 | 45 ± 10 |
| *Fractal dimension* | 1.37 ± 0.02 | 1.35 ± 0.04 | 1.36 ± 0.04 | 1.30 ± 0.06 | 1.36 ± 0.03 | 1.28 ± 0.06 |
| *Lacunarity* | 0.41 ± 0.05 | 0.38 ± 0.04 | 0.42 ± 0.05 | 0.38 ± 0.05 | 0.42 ± 0.07 | 0.37 ± 0.07 |

**TABLE S.3.** Morphometric values of microglial cells from hippocampus (mean ± SD, n=155)

|  | **2 h** | | **4 h** | | **12 h** | |
| --- | --- | --- | --- | --- | --- | --- |
| **Parameter** | **Saline** | **NA** | **Saline** | **NA** | **Saline** | **NA** |
| *Cell circularity* | 0.003 ± 0.001 | 0.004 ± 0.002 | 0.003 ± 0.001 | 0.013 ± 0.008 | 0.003 ± 0.001 | 0.018 ± 0.020 |
| *Convex hull area* (µm^2^) | 3107 ± 697 | 2926 ± 719 | 3416 ± 702 | 1640 ± 748 | 3235 ± 642 | 1422 ± 635 |
| *Cell perimeter* (µm) | 1687 ± 383 | 1662 ± 401 | 1929 ± 386 | 844 ± 370 | 1827 ± 426 | 729 ± 329 |
| *Convex hull span ratio* | 1.4 ± 0.2 | 1.3 ± 0.2 | 1.4 ± 0.2 | 1.5 ± 0.6 | 1.3 ± 0.1 | 1.5 ± 0.3 |
| *Convex hull circularity* | 0.87 ± 0.04 | 0.87 ± 0.04 | 0.87 ± 0.03 | 0.82 ± 0.10 | 0.88 ± 0.03 | 0.82 ± 0.07 |
| *Max/Min convex hull radii* | 1.6 ± 0.2 | 1.6 ± 0.2 | 1.6 ± 0.2 | 1.9 ± 0.6 | 1.5 ± 0.2 | 1.9 ± 0.5 |
| *Roughness* | 8.0 ± 1.1 | 8.1 ± 1.5 | 8.7 ± 1.2 | 5.3 ± 1.6 | 8.4 ± 1.3 | 4.9 ± 1.5 |
| *Convex hull perimeter* (µm) | 210 ± 24 | 204 ± 26 | 221 ± 23 | 155 ± 33 | 214 ± 22 | 143 ± 34 |
| *Density* | 0.21 ± 0.04 | 0.26 ± 0.05 | 0.22 ± 0.04 | 0.34 ± 0.09 | 0.21 ± 0.03 | 0.35 ± 0.08 |
| *Convex hull mean radii* (µm) | 35 ± 4 | 34 ± 4 | 37 ± 4 | 26 ± 6 | 35 ± 4 | 24 ± 5 |
| *Cell area* (µm^2^) | 651 ± 183 | 754 ± 199 | 747 ± 126 | 533 ± 198 | 686 ± 171 | 494 ± 238 |
| *Diameter of the bounding circle* (µm) | 78 ± 9 | 75 ± 9 | 82 ± 10 | 56 ± 12 | 79 ± 9 | 55 ± 14 |
| *Maximun span across the convex hull* (µm) | 78 ± 10 | 74 ± 9 | 81 ± 10 | 58 ± 12 | 78 ± 9 | 54 ± 14 |
| *Fractal dimension* | 1.39 ± 0.03 | 1.40 ± 0.03 | 1.42 ± 0.04 | 1.33 ± 0.05 | 1.41 ± 0.04 | 1.32 ± 0.05 |
| *Lacunarity* | 0.41 ± 0.06 | 0.39 ± 0.06 | 0.46 ± 0.09 | 0.38 ± 0.05 | 0.44 ± 0.06 | 0.38 ± 0.06 |

**TABLE S.4.** Morphometric values of microglial cells from hypothalamus (mean ± SD, n=155)

|  | **2 h** | | **4 h** | | **12 h** | |
| --- | --- | --- | --- | --- | --- | --- |
| **Parameter** | **Saline** | **NA** | **Saline** | **NA** | **Saline** | **NA** |
| *Cell circularity* | 0.010 ± 0.004 | 0.018 ± 0.010 | 0.009 ± 0.004 | 0.028 ± 0.009 | 0.008 ± 0.002 | 0.032 ± 0.019 |
| *Convex hull area* (µm^2^) | 1395 ± 427 | 1006 ± 303 | 1626 ± 591 | 763 ± 216 | 1589 ± 445 | 777 ± 429 |
| *Cell perimeter* (µm) | 773 ± 188 | 558 ± 158 | 795 ± 230 | 411 ± 88 | 797 ± 193 | 431 ± 289 |
| *Convex hull span ratio* | 1.4 ± 0.2 | 1.5 ± 0.3 | 1.7 ± 0.4 | 1.5 ± 0.3 | 1.5 ± 0.3 | 1.6 ± 0.3 |
| *Convex hull circularity* | 0.85 ± 0.04 | 0.81 ± 0.06 | 0.78 ± 0.08 | 0.82 ± 0.05 | 0.82 ± 0.06 | 0.80 ± 0.06 |
| *Max/Min convex hull radii* | 1.8 ± 0.3 | 1.9 ± 0.3 | 2.0 ± 0.5 | 1.7 ± 0.3 | 1.8 ± 0.4 | 2.1 ± 0.5 |
| *Roughness* | 5.4 ± 1.0 | 4.5 ± 0.8 | 4.9 ± 0.9 | 3.8 ± 0.5 | 5.1 ± 1.0 | 3.8 ± 1.6 |
| *Convex hull perimeter* (µm) | 143 ± 22 | 123 ± 18 | 159 ± 26 | 107 ± 15 | 154 ± 22 | 107 ± 27 |
| *Density* | 0.31 ± 0.05 | 0.38 ± 0.06 | 0.26 ± 0.05 | 0.47 ± 0.06 | 0.26 ± 0.07 | 0.43 ± 0.10 |
| *Convex hull mean radii* (µm) | 24 ± 4 | 21 ± 3 | 27 ± 5 | 18 ± 3 | 26 ± 4 | 18 ± 5 |
| *Cell area* (µm^2^) | 422 ± 91 | 370 ± 89 | 411 ± 124 | 356 ± 91 | 410 ± 164 | 306 ± 119 |
| *Diameter of the bounding circle* (µm) | 54 ± 8 | 47 ± 8 | 63 ± 12 | 41 ± 6 | 59 ± 9 | 42 ± 10 |
| *Maximun span across the convex hull* (µm) | 53 ± 8 | 47 ± 8 | 62 ± 12 | 41 ± 6 | 59 ± 10 | 42 ± 10 |
| *Fractal dimension* | 1.35 ± 0.02 | 1.32 ± 0.04 | 1.33 ± 0.03 | 1.29 ± 0.04 | 1.33 ± 0.03 | 1.27 ± 0.04 |
| *Lacunarity* | 0.37 ± 0.03 | 0.36 ± 0.03 | 0.41 ± 0.05 | 0.34 ± 0.04 | 0.40 ± 0.05 | 0.37 ± 0.04 |
